# Understanding further education as a context for public health intervention: qualitative findings from a study process evaluation

**DOI:** 10.1093/pubmed/fdz059

**Published:** 2019-06-04

**Authors:** R Langford, M Willmott, A Fletcher

**Affiliations:** 1 Population Health Sciences, Bristol Medical School, University of Bristol, Oakfield House, Oakfield Road, Bristol, UK; 2 Department of Public Health, Environments and Society, London School of Hygiene and Tropical Medicine, 15–17 Tavistock Place, London, UK

**Keywords:** Further education, intervention, process evaluation, public health, qualitative research, young people

## Abstract

**Background:**

Over 1.2 million 16–18 year-olds are enrolled in further education (FE—advanced secondary education) in England. Life course transitions provide opportunities to change, establish or reinforce health behaviours. FE presents an opportunity for public health improvement, yet few interventions target this setting. Using a smoking prevention intervention, we explore how young people were viewed in FE and how this affected intervention acceptability.

**Methods:**

Eleven student and five staff focus groups were conducted in three intervention institutions (two colleges, one school sixth-form), as part of the process evaluation of a smoking prevention feasibility study. FE managers in intervention and control institutions were also interviewed (*n* = 5). Data were analysed using thematic analysis.

**Results:**

In both colleges and the sixth-form, students were viewed as emergent adults and treated differently from ‘school-children’, in practice if not in policy. Colleges permitted smoking in designated areas; in the school sixth-form smoking was unofficially tolerated but concealed from younger students. Using staff to deliver anti-smoking messages reintroduced an unwanted power dynamic which disrupted perceptions of students as young adults.

**Conclusions:**

FE is an important setting for young people’s health. Understanding the culture and context of FE is critical in designing acceptable and effective public health interventions.

## Introduction

Over 1.2 million 16–18 year olds are enrolled in further education (FE) courses in England,^1^ most commonly in FE colleges or ‘sixth-forms’ located within schools (Box 1). Life course transitions provide opportunities to change, establish or reinforce health behaviours^2–4^; the transition from secondary school to FE thus offers an important opportunity for public health intervention. However, little evidence exists to inform such interventions. There are no published systematic reviews of health interventions in FE, and only 9% (21/246) of studies included in four Cochrane reviews of interventions in educational settings involved 16–18 year-olds.^5–8^ Consequently, little is known about how to promote health in FE settings.

Box 1 Further Education (FE) in the UK

**Table:** 

Young people aged 16–18 years in the UK are legally bound to be in education, employment or training. FE refers to education provided to people over the age of 16 in the UK. It is distinct from higher education (HE) which is usually offered in universities (although FE institutions may provide HE courses). FE may be delivered in public and privately funded institutions, most commonly in schools (often in separate units called ‘sixth-forms’) or FE colleges. Courses delivered in FE colleges are typically more vocational than those offered in school sixth-forms.FE is predominantly a UK phenomenon but a similar secondary and advanced secondary education system exists in the Republic of Ireland. To aid understanding, we suggest that international equivalents in other high income countries are Technical and Further Education (TAFE) in Australia and ‘continuing education’ in North America, although both of these primarily provide vocational courses, unlike FE in the UK which provides academic courses too.

While there is an extensive literature on school-based (<16 years) interventions^5–11^ and a growing evidence base for Higher Education (university) settings,^12,13^ FE has been neglected. Recent guidance has highlighted the importance of understanding ‘context’ in determining ‘*how* [interventions] *work* [and] *why they sometimes fail*’(14:viii). Context is a multi-faceted concept encompassing numerous domains including social, political and organisational factors,^14^ relationships between actors, perceptions of social norms, and local community characteristics.^15^ Context is thus ‘*a constellation of active interacting variables and is not just a backdrop for implementation’* (16:50).

It is naive to assume what works in schools or universities will be equally successful in FE, without exploration of the contextual attributes that make FE distinctive, and how these may vary by provider type (e.g. college vs. school sixth-form, Box 1). Without this contextual understanding, public health interventions in FE may be inappropriate, unacceptable or under-theorized, and therefore less effective.^17,18^

Drawing on a smoking prevention feasibility study conducted in FE, we discuss how FE policies and practices - and student and staff responses to these – provide insight into FE as a context for public health intervention.

### Smoking and The Filter FE intervention

Nearly half (44%) of regular smokers start to smoke between 16 and 19 years.^19^*The Filter FE* was a multicomponent smoking prevention intervention^20^ targeting 16–18 year-olds in FE settings (Box 2). Developed in collaboration with ASH Wales, it adapted an existing programme called *The Filter*,^21^ working at community, institutional and individual levels to prevent uptake of smoking in this population (on-line appendix, logic model). To provide background, within the UK it is illegal to sell tobacco to anyone under 18 years, but there is no legal age restriction on smoking tobacco.

Box 2 The Filter FE challenge

**Table:** 

The intervention was delivered by ASH Wales, drawing on their existing youth smoking prevention resources and applying them in further education (FE) settings. It aimed to prevent smoking uptake among FE students aged 16–18 via: (1) restricting the sale of tobacco to under 18 year-olds; (2) implementing tobacco-free campus policies; (3) training FE staff to deliver anti-smoking messages and support institutional change; (4) publicizing ‘The Filter’ youth project’s online social marketing campaigns, advice and support services; and (5) youth work activities to provide credible educational messages, address norms, promote resistance skills and signpost cessation services.

Data for this paper are drawn from the process evaluation of a feasibility randomized controlled trial (RCT) testing *The Filter FE*’s fidelity, feasibility and acceptability. The full evaluation is reported elsewhere.^20^ We limit our discussion here to the institutional elements of the intervention as these generated most discussion by students and staff and offered greatest insight into FE context. The institutional elements aimed to: (i) implement campus-wide tobacco-free policies and (ii) provide staff training to deliver smoking prevention messages to students.^20^ We used the data generated to explore how young people were viewed in FE and how this affected intervention acceptability.

## Methods

Focus groups and semi-structured interviews were conducted (by MW/RL/AF) at the end of the intervention, as part of *The Filter FE* process evaluation. Six FE institutions in Wales were stratified and matched by size (Table 1) and randomized to receive *The Filter FE* intervention or continue with usual practice.^20^ Data were collected from focus groups in the three intervention institutions and manager interviews at both intervention and control institutions. Focus groups facilitated interaction between participants, allowing us to collect rich data from a large number of participants^22^; interviews (rather than focus groups) were conducted with FE managers for pragmatic reasons.

**Table 1:** Study site characteristics and data collected

FE Institution pseudonym	Group	Setting characteristics*	Staff focus groups (N)	Student focus groups (N)	FE Manager interviews (N)
Valeside College	INTERVENTION	Large FE college	2	5	1
Laurelton College	INTERVENTION	Small FE college	2	4	1
Athervale School	INTERVENTION	School sixth-form	1	2	1
Middledale College	CONTROL	Large FE college	–	–	1
Glynbel College	CONTROL	Small FE college	–	–	1
Afonwood School	CONTROL	School sixth-form	–	–	0

FE = Further Education. *Size determined by new intake of students per year: <500 students = small; >500 students = large.

**Table 2:** Student/staff focus group and Further Education (FE) Manager interview topics

**Student & Staff focus group topics**
Student smoking in your sixth-form/college—who, when, where, why? The Filter FE intervention – views on intervention elements (restricting tobacco scales, smoke-free campus policies, staff training, youth work, social media) Recruiting and collecting data from students
**FE Manager interview topics**
Decision to participate in research For intervention sites only: – Implementation of *The Filter FE* intervention – Perceived impact of the intervention For control sites only: – What is ‘usual practice’ (existing policies or training on smoking)? – Has taking part in the research changed anything (e.g. changed policies, raised awareness?)?

Focus groups in the three intervention settings explored participants’ views of the intervention, and their perceptions and experiences of smoking in a FE context (Table 2). Eleven student focus groups were conducted (*n* = 69 participants, range 2–13 per group) using convenience samples of participants recruited via friendship or tutorial groups (for institution recruitment, see^20^). They lasted approximately 45 minutes and included full- and part-time students on academic and vocational courses. Forty-five percent of participants (31/69) were female, and a quarter (18/69) identified as smokers. We were unable to attribute quotations to specific individuals to identify gender or smoking status. Five staff focus groups were conducted across intervention sites (*n* = 19 participants, range 2–6 per group). Participants represented a range of teaching and support positions; demographic and smoking status data were not collected.

Semi-structured interviews were conducted with FE managers in both intervention and control institutions (*n* = 5, one manager declined). They were conducted either face-to-face or over the telephone, according to participants’ preference; most lasted about 30 minutes.

Focus groups and interviews were recorded, transcribed verbatim and entered into NVivo 10™ software. An iterative coding framework for thematic analysis,^23^ developed by MW from initial reading of two transcripts, was subsequently independently applied to three further transcripts by MW and RL to ensure consistency. MW applied the agreed framework to all manuscripts with modifications discussed between authors. Relationships between themes and variations between groups were scrutinized throughout. Cardiff University’s School of Social Sciences Research Ethics Committee provided ethical approval. College/sixth-form names are pseudonyms.

## Results

### Smoking policies

Both intervention colleges had established policies permitting smoking only in designated smoking areas (DSAs); the intervention school sixth-form had a blanket no-smoking policy.

In general, college students and staff felt the policy of only smoking in designated areas was well-accepted and respected. There appeared little enthusiasm among students or staff for a campus-wide smoking ban, as intended by *The Filter FE* intervention. Staff viewed DSAs as a pragmatic solution, arguing students would *‘find somewhere, so it’s controlled to an extent’* (Laurelton College staff). College policies of DSAs appealed to students because it embodied the autonomy they associated with a non-school environment. One staff member explained: ‘*We used to take school groups around* […] *one of the first questions a schoolchild would ask, ‘are you allowed to smoke in college?’* […] *we don’t have uniform, it was a freer environment*’ (Valeside College staff). Reinforcing this, a sixth-form student said, ‘*up the college they’ve got a bus stop which is a smoking area*… *that’s the thing I like about college*’ (Athervale sixth-form student).

While college staff and students were aware of the health implications of smoking, competing priorities limited the acceptability of a total smoking ban. Staff wanted to protect their institutional reputation, acknowledging students smoking just outside campus *‘would look quite bad for the college’* (Laurelton College Staff). Allowing DSAs on campus thus rendered student smoking less visible to the wider community, as one FE manager explained:

*‘We’ve got a smoking shelter at the back, which I think we need to keep really, because otherwise they’re going to be [smoking] out on the street in front of people’s houses, which isn’t nice. Or they go down the park, and again, that’s not nice… So, it’s not really possible for us to have a smoke-free environment.*’ (Valeside College Manager)

Staff also cited their duty of care towards students to justify their institution’s smoking policy. A smoking ban had been considered by Laurelton College but the ‘[college authorities] *thought the students would then go on to the main road*’ potentially increasing the risk of traffic accidents. Another staff member suggested, *‘if you prohibit it completely, people will find a dark cubby-hole somewhere and there’ll be more risk of fires.*’

By contrast, the blanket ban enforced at the sixth-form frustrated students: *‘we’re not allowed out of school but we’re not allowed to smoke in school, so where are we supposed to go?’* (Athervale sixth-form student). The presence of younger students at the school appeared to influence official policy and its enforcement. Providing DSAs was not acceptable as it might *‘encourage* [younger] *students to join them’* (Athervale sixth-form staff). However, staff appeared to tacitly accept it was less legitimate to control sixth-form students’ smoking behaviour. As one student explained, ‘*the teachers are more laid back about* [smoking] *because we’re old enough’.* Thus, despite the official policy, an *‘informal designated area*’ existed, referred to by staff as *‘Smokers’ corner.’* Staff acknowledged *‘all we do is contain it really’* and explained they ‘*usher*’ smokers to a secluded area away from school buildings and younger students.

School sixth-form staff tolerated transgressions of the smoking policy for similar reasons to those given by college staff for not pursuing a smoking ban: their duty of care and concern for institutional reputation. Sixth-form staff acknowledged ‘[we] *can’t send them off the premises, because obviously they’re under our care aren’t they?’* and recognized that rigorously enforcing rules could push smokers off site. One-sixth-form staff member raised concerns about student safety if they left the premises – ‘*if they got knocked over by a bus on the way back, no-one would know would they?’* – while another felt it would impact on their learning time. Students also recognised the reputational impact of smoking off site, explaining, ‘[teachers] *don’t like people smoking at the shop because it still looks bad, like a bunch of school girls’* (Athervale sixth-form student).

### Smoking prevention messages

While staff were asked to deliver smoking *prevention* messages, most participants highlighted the inappropriateness of telling smokers to stop smoking. Students and staff perceived most smokers had started before arriving in FE, rendering prevention messages redundant. For context, 21% students in the trial were weekly smokers at baseline; due to loss-to-follow-up, we were unable to calculate the percentage initiating smoking during the intervention period.^20^

Students and staff viewed smoking as an individual, self-determined choice. As one student stated, ‘*If you want to do it, crack on… whatever people want to do, it’s fine by me*.’ (Valeside College student), while a staff member commented, *‘It’s their choice to smoke… we don’t preach to them’* (Laurelton College staff). Staff were more concerned about ensuring students smoke *‘in the correct way’* (i.e. legal substances and in designated areas) and providing relevant information: *‘these are the facts, this is what happens, and these are the people who can help you’* (Laurelton College staff).

Building on both their belief that smoking is an individual choice and their self-image as young adults, college students questioned the legitimacy of staff attempting to restrict or comment on their behaviour. One student argued, ‘*most of the people here are over 18 so it’s just telling a grown up not to do something. It’s a bit weird, isn’t it?*’ (Valeside College student). Staff agreed, explaining students would find it *‘condescending’* to be lectured about quitting: ‘*they’re old enough to get married, they’re old enough to do other things. I think they wouldn’t appreciate it.’* (Laurelton College staff).

Ultimately, the primary responsibility of FE staff is to educate, and this appeared to influence staff responses to student smoking. Some staff spoke of the addictive nature of smoking and recognised the detrimental impact it could have on learning and behaviour. As one commented, *‘some of them obviously need to have a smoke. You can see them getting all pent up, and I keep saying, why don’t you get a patch when you’re in school?’* (Athervale sixth-form staff). This impact was acknowledged by students too who argued they concentrated better after a cigarette.

Staff were also aware their ability to teach required positive staff-student relationships, and sanctioning students about smoking could threaten this. One staff member explained she did not discuss students’ smoking with parents ‘*because if they don’t get that bond with us, they’re not going to interact with us or trust us’* (Laurelton College staff), while a sixth-form teacher described the *‘abuse’* she would get for reporting students’ smoking, with another adding *‘it’ll impact into your lessons as well if you teach them’* (Athervale sixth-form staff).

Nonetheless there were some positive examples of staff challenging students’ smoking. Staff suggested these conversations needed to be handled carefully – *‘we can’t do it in such a way that we’re preaching because they won’t come to us then’* (Laurelton College Staff) – and occurred only when a strong relationship was established: ‘W*e’re quite friendly with our teachers, we can have a bit of a joke and we get along, we’ve known each other for a couple of years. There’s a kid in my class who constantly has to have a fag all the time… So the teachers joke about it… it’s more of a friendly thing’* (Valeside College student). It appeared more difficult to have these conversations at the sixth-form, with students suggesting ‘*I don’t think they would listen*’ to teachers. One-sixth-form staff member echoed this explaining, ‘[when I was young] *I wouldn’t have listened to my mother, I wouldn’t listen to a teacher*’ (Athervale sixth-form staff), while another suggested students would prefer messages to come from ‘*someone outside of the school*’.

## Discussion

### Main findings

Student and staff responses to smoking provide insight into the way young people are viewed in FE: as emergent adults, different from school students (under 16 years) and therefore treated differently (in practice, if not in policy). Although staff justified their stance on smoking by invoking similar principles (e.g. protecting institutional reputation, ensuring student safety), smoking policies and staff-student interactions played out differently in the sixth-form compared with colleges. This appeared to relate to the wider student population within the setting; school-based sixth-forms needed to shield younger students, despite informal smoking areas being tacitly accepted. Conversely, the presence of over-18 s in colleges made placing limits on smoking behaviour less acceptable within this more adult-orientated context.

In recognition of FE students’ growing autonomy and perceived move to adulthood, staff and students were reluctant to discuss smoking because it disrupted their mutual perception of students as young adults and potentially reintroduced a power dynamic associated with younger age groups. Though there were occasions where staff reported talking to students about smoking, these occurred within an established, friendly relationship. Such conversations were more difficult in the sixth-form where more formal relationships between staff and student appeared to be maintained.

### What is already known?

Few smoking interventions have been conducted in FE settings. A quasi-experimental study in vocational training colleges in France provided students with health education, counselling and nicotine-replacement therapy and was found to be effective in supporting smoking cessation.^24^ Motivational interviewing provided to UK FE students was effective at reducing tobacco, alcohol and drug consumption in one RCT,^25^ but ineffective at preventing initiation in another.^26^ Importantly, in all three cases, external actors provided intervention activities, not FE staff. Unlike *The Filter FE* there was no attempt to modify the FE context in terms of policies or practices.

The wider evidence base for health promotion in FE settings is weak: we found no other RCTs in this setting on any health topic but did identify cross-sectional surveys and qualitative studies focusing on mental health,^27–30^ substance use,^31^ social norms,^32^ driving behaviour,^33^ food behaviours,^34^ relationship violence^35^ and sexual health.^36^ Research evidence and theories attest to the power of education and educational settings as a wider determinant of health, albeit via complex and often under-researched mechanisms.^18,37^ In addition, while not focusing specifically on FE, a systematic review of the health effects of the school environment suggests implementation of interventions may be facilitated when they build on existing ethos.^38^

### What this study adds

This is one of the first qualitative studies from a public health RCT in FE settings. It provides insight into the context of an under-researched but potentially fruitful setting for public health intervention.

FE is often characterised by greater informality and equality between educators and students than in educational settings for younger age groups. This is often visibly demonstrated through the lack of uniform and use of staff first names.^39^ This ‘FE ethos’, recognizing students’ emergent adulthood, is highly valued, particularly by younger students entering FE for the first time^39–42^ and by staff who value the positive learning culture it allows them to create.^42^ School-health theorists have argued that breaking down staff-student boundaries creates environments conducive to better student (and staff) health and well-being.^18,43^ The more egalitarian ethos within FE may therefore provide a positive foundation on which to base public health intervention.

However, our study found the liberal ethos of FE which treats students as autonomous adults fitted uncomfortably with *The Filter FE*’s smoking prevention messages, with staff and students echoing tobacco industry rhetoric around individual choice.^44^ The continued strength of this rhetoric is disappointing, but not surprising, given the association between smoking and identity formation,^45^ the liminal legal and social status of 16–18 year-olds^46^ and the specific targeting of young people undergoing life course transitions.^47^That some staff were able to comfortably discuss smoking with students with whom they had good relationships suggests this perception can be challenged though. Further exploration of the supportive relationships between FE staff and students, and how public health could capitalize on these, would be of benefit.

### Limitations

Our qualitative data generated in-depth insights into FE as a site for public health intervention. Data were collected from three FE institutions, which differed in nature and size. As FE is a heterogeneous sector, our findings require further exploration in other FE institutions. We used convenience sampling to recruit our focus groups, meaning their representativeness is unknown. For students, we attempted to recruit smokers and non-smokers into separate groups. However, students did not always identify with those labels. Several groups therefore contained a mix of smokers and non-smokers which may have affected what students said. It also means it was not possible to attribute quotations to students according to their smoking status. Semi-structured interviews may have elicited more detailed information from participants than focus groups, but were not possible due to resource constraints. Data collected for this study focused on the topic of intervention, namely smoking prevention. Further research could explore how themes identified here manifest across different public health topics in this context.

## Conclusions

Existing at a transitional moment in young people’s lives, FE represents an important context for public health, yet little is known on how best to intervene here. Future research should explore this context in greater depth, to better understand relationships between staff and students, perceived health needs, and internal and external influences on FE as an intervention setting.

## Supplementary Material

fdz059_Online_appendix,_intervention_logic_model
