# Supplementary figures and images for: Understanding further education as a context for public health intervention: qualitative findings from a study process evaluation
Source: J Public Health (Oxf). 2019 Jun 4;42(3):610–7. doi: 10.1093/pubmed/fdz059 (PMC7435218; doi:10.1093/pubmed/fdz059)

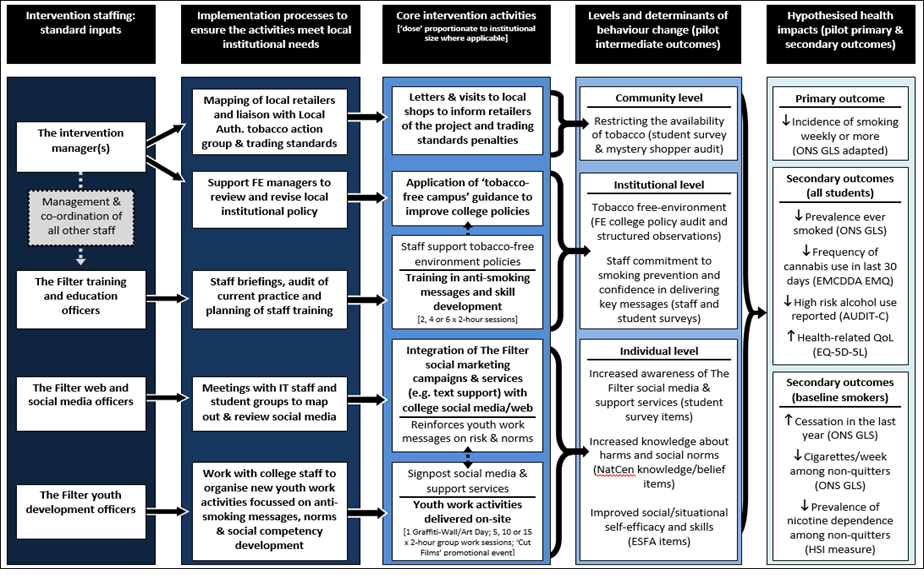

Supplement: fdz059_Online_appendix,_intervention_logic_model [file fdz059_online_appendix,_intervention_logic_model.png]
